# Area-Specific Alterations of Synaptic Plasticity in the 5XFAD Mouse Model of Alzheimer’s Disease: Dissociation between Somatosensory Cortex and Hippocampus

**DOI:** 10.1371/journal.pone.0074667

**Published:** 2013-09-17

**Authors:** Nadine Crouzin, Kevin Baranger, Mélanie Cavalier, Yannick Marchalant, Catherine Cohen-Solal, François S. Roman, Michel Khrestchatisky, Santiago Rivera, François Féron, Michel Vignes

**Affiliations:** 1 Laboratory UMR7259 ‘Neurobiologie des Interactions Cellulaires et Neurophysiopathologie’, Aix-Marseille University, Marseille, France; 2 Laboratory UMR7259 ‘Neurobiologie des Interactions Cellulaires et Neurophysiopathologie’, CNRS, Marseille, France; 3 Laboratory UMR5247 ‘Institut des Biomolécules Max Mousseron’, University of Montpellier 1, University of Montpellier 2, CNRS, Montpellier, France; Torrey Pines Institute for Molecular Studies, United States of America

## Abstract

Transgenic mouse models of Alzheimer’s disease (AD) that overproduce the amyloid beta peptide (Aβ) have highlighted impairments of hippocampal long-term synaptic plasticity associated with the progression of the disease. Here we examined whether the characteristics of one of the hallmarks of AD, i.e. Aβ deposition, in both the somatosensory cortex and the hippocampus, correlated with specific losses of synaptic plasticity in these areas. For this, we evaluated the occurrence of long-term potentiation (LTP) in the cortex and the hippocampus of 6-month old 5xFAD transgenic mice that exhibited massive Aβ deposition in both regions but with different features: in cortical areas a majority of Aβ deposits comprised a dense core surrounded by a diffuse corona while such kind of Aβ deposition was less frequently observed in the hippocampus. In order to simultaneously monitor synaptic changes in both areas, we developed a method based on the use of Multi-Electrode Arrays (MEA). When compared with wild-type (WT) mice, basal transmission was significantly reduced in both areas in 5xFAD mice, while short-term synaptic plasticity was unaffected. The induction of long-term changes of synaptic transmission by different protocols revealed that in 5xFAD mice, LTP in the layer 5 of the somatosensory cortex was more severely impaired than LTP triggered in the CA1 area of the hippocampus. We conclude that cortical plasticity is deficient in the 5xFAD model and that this deficit could be correlated with the proportion of diffuse plaques in 5xFAD mice.

## Introduction

Alzheimer’s disease (AD) is a neurodegenerative disorder associated with progressive cognitive decline and extensive neuronal loss [1]. Numerous alterations within the brain of AD patients have been identified after post-mortem analyses: brain deposition of senile plaques containing Aβ peptide, intracellular neurofibrillary tangles of hyperphosphorylated tau protein, reduced synaptic density, neuro-inflammation, and extensive cell death in different structures critically involved in cognitive functions such as learning and memory [2]. In this line, the introduction of mutant forms of human amyloid protein precursor (APP), presenilin (*PSEN*) and/or *tau* genes in mice reproduce many pathological features of the disease, such as Aβ deposits, neurofibrillary tangles, gliosis, and synaptic degeneration. At the cognitive level, the majority of these AD mouse models exhibit memory deficits demonstrated by their poor performances in the Morris’ water-maze, Y-maze, fear conditioning task, and object or social recognition tasks [3,4]. Although to date no perfect model of AD has emerged, transgenic mice carrying the mutated human *APP*, *PSEN1* and *tau* genes or combining more than one of these mutations successfully recapitulate most of AD markers [5]. Amongst all transgenic mice that have been developed, 5xFAD mice (Tg6799 line) harboring five familial AD (FAD) mutations (3 on human *APP* and 2 on *PSEN1*) represent one of the most early-onset and aggressive amyloid mouse models of AD [6-8]. Indeed, while it takes at least 6–12 months to form Aβ plaques in the majority of AD transgenic mice (Eriksen and Janus, 2007), 5xFAD mice start to develop visible Aβ deposits as early as 2 months of age, consistent with their dramatically accelerated Aβ1-42 production. This Aβ deposition first emerges in the subiculum area of the hippocampus and in the cortical layer 5, and then rapidly increases with age, spreading to fill much of the hippocampus and cortex by 9 months of age [7]. 5xFAD mice also exhibit memory dysfunctions as highlighted using numerous behavior tests such as Y-maze and water-maze which characterize hippocampus-dependent cognitive processes [7,9] as well as conditioned taste aversion, contextual fear conditioning and H-maze which are characteristic of cognitive processes dependent on cortex [10-13]. Hippocampus-dependent deficits in 5xFAD mice, as in numerous other AD models [14], can be explained by deficits in hippocampal synaptic plasticity as evidenced by impairments of LTP, the molecular substrate of learning and memory [6,15]. However, there are accumulating evidences that memories of everyday life may initially depend on the hippocampus, but are not instantaneously formed and have to undergo a subsequent prolonged period of reorganization [16-19]. In particular, as memories mature, they become increasingly independent of the hippocampus and memory traces are gradually stabilized and eventually transformed into remote memories localized in cortical networks. Importantly, significant Aβ accumulation occurs not only in the hippocampus but also in the cerebral cortex of AD patients and of transgenic mouse models of AD [14,20] and almost all learning and memory processes, including long-term as well as short-term memories, are impaired in AD [21,22]. Aβ depositions are associated with strong neuroinflammation as evidenced by the occurrence of pro-inflammatory cells (microglia and astrocytes) around these depositions [7]. In fact, neuroinflammation was shown to be a modulatory factor of synaptic plasticity [23].

Therefore, in line with this, we have tested here whether the extent of Aβ depositions and the phenotypic differences across plaques in both cortex and hippocampus could correlate with specific losses of synaptic plasticity in these areas. For this, we have evaluated the occurrence of LTP in the cortex and the hippocampus of 6-month old 5xFAD mice that showed massive Aβ deposition in cortical area and in the CA1 area of hippocampus at this age with subtle differences in phenotypes of plaques (dense core with or without diffuse corona according to the area considered). Moreover, in order to improve temporal and regional correlations in our recordings, we developed a method based on the use of Multi-Electrode Arrays (MEA), which allowed the simultaneous monitoring of plasticity in the layer 5 of the somatosensory cortex (SSC) and the CA1 area of the hippocampus.

## Materials and Methods

### Animals

All experiments were carried out in accordance with the European Community Council Directive of November 24, 1986 (86/609/ECC). This study was approved by the local branch of the ‘Comité National de Réflexion Ethique sur l’Expérimentation Animale’ (C2EA-36). All efforts were made to minimize animal suffering and to reduce the number of mice used. The generation of 5xFAD mice has been previously described [7]. These transgenic mice over-produce both mutant human *APP* (695) with the Swedish (K670N, M671L), Florida (I716V), and London (V717I) Familial Alzheimer’s Disease (FAD) mutations and human *PSEN1* harboring two FAD mutations, M146L and L286V. Expression of both transgenes is regulated by the neuronal-specific Thy1 promoter. The 5xFAD strain (B6/SJL genetic background) was maintained by crossing hemizygous transgenic mice with B6/SJL F1 breeders (Jackson Laboratories, Bar Harbor, Maine, USA). Non-transgenic WT littermate mice were used as controls. All transgenic and WT mice were bred in our animal facility, had access to food and water *ad libitum*, and were housed under a 12 h light-dark cycle at 22–24°C.

### Immunostaining and quantification of Aβ deposits

Mice were deeply anesthetized (sodium pentobarbital, *i.p.*) and transcardially perfused with 4% paraformaldehyde. Brains were dissected and post-fixed overnight in 4% paraformaldehyde at 4°C. Sagittal brain sections (30 µm thick) were serially cut using a vibratome (Thermo Scientific HM650V, Illkirch, France) and stored at −20°C in 6-well plates containing a cryoprotective solution (30% glycerol, 30% ethylene glycol in 0.05 M PBS) until processed for immunostaining. After washing in PBS, floating sections were incubated 1 h at room temperature (RT) with blocking buffer (3% BSA, 0.1% Triton X-100 in PBS) and then overnight at 4°C with the primary antibodies i.e. mouse monoclonal anti-Aβ 6E10 (1/200, Covance (Eurogentec), Angers, France) diluted in blocking solution. Then, slices were rinsed (3×5min) in PBS and incubated for 90 min at RT with cross-adsorbed Alexafluor 488 (green)- or 594 (red)-conjugated anti-mouse secondary antibody (1/500, Jackson Immunoresearch, West Grove, PA, USA) in dark conditions. After several washes in PBS, slices were counter-stained with 0.5 µg/ml Hoechst blue (#33342, Sigma-Aldrich, Saint-Quentin Fallavier, France) for 30 min at RT and mounted with ProLong Gold Antifade reagent (Life Technologies, Saint-Aubin, France).

Images were acquired using a Zeiss inverted Axio Observer microscope (Zeiss, Jena, Germany). Images of large brain sections were obtained using the MosaiX mode of the Axiovision software (Zeiss). Number and type (i.e. dense core *versus* diffuse plaques) of amyloid deposits in the cortex and the hippocampus were manually counted and the number of plaques per mm^2^ of brain surface was calculated using Axiovision software (Zeiss).

### Electrophysiological recordings with micro-electrode array

Experiments were carried out on brain slices containing both hippocampus and cortex (300 µm thickness) obtained from 6 month-old male mice. After decapitation, brains were quickly dissected and placed in ice-cold buffer comprising 124 mM NaCl, 3.5 mM KCl, 25 mM NaHCO_3_, 1.25 mM NaH2PO_4_, 1 mM CaCl_2_, 2 mM MgSO_4_, 10 mM D-glucose, and 10 mM HEPES (bubbled with O_2_/CO_2_, 95/5). Slices were then cut with a Vibratome (VT1000S; Leica, France) and maintained at RT for at least 1 h in the same buffer supplemented with 1 mM CaCl_2_. This supplemented buffer – also named extracellular medium - was used for further recordings.

For electrophysiological recordings, slices were transferred to a MEA (MEA60; Multi Channel Systems, Reutlingen, Germany) continually superfused with the above described extracellular medium (flow rate 2 ml.min^−1^) and kept at 32°C. Drugs were directly applied in this superfusion. MEA was positioned on the platform of a Leica inverted microscope equipped with a CCD camera (CoolSnap, Roper Scientific, France). Images of the cortico-hippocampal slice on the MEA were captured in order to accurately map the synaptic signals recorded in the different areas of the brain slice. MEAs comprised 60 extracellular electrodes [24]. The inter-electrode distance was 200 µm. Each individual electrode from the array could be used either as a recording or as a stimulatory electrode. A nylon mesh was positioned above the slice to obtain a satisfactory electrical contact between the surface of the slice and the electrode array. Double stimulation was achieved with an external stimulator (STG-1004; Multi Channel Systems) by applying biphasic current pulses simultaneously to two electrodes of the array, one located in the Schaffer Collateral pathway of the hippocampus and the other one in the cortex. Stimulation intensity (60 to 300 µA) and duration (70 to 200 µs) were adapted to avoid multiphasic responses due to an excessive stimulation [25]. Field excitatory postsynaptic potentials (fEPSPs) and population spikes (pop spikes) could then be recorded by all the remaining electrodes of the array at the same time. Signals were recorded and analyzed (MC Rack; Multi Channel Systems). We verified that stimulating cortical afferents did not evoke any signal in the stratum radiatum of the CA1 area of the hippocampus and conversely that stimulating Schaffer collaterals pathway did not induce any signal in the cortical area recorded. In fact, both hippocampal and/or cortical stimulation could evoke some signals in the Alveus. Thus these signals were not taken into consideration for further analysis. Baseline synaptic signals were evoked using a 0.066 Hz frequency. Slices displaying epileptic-like activity were discarded. Short term plasticity was elicited by two stimulations with an interpulse duration varying from 25 to 500ms. Long-term potentiation (LTP) was induced using one or three repeated theta-burst stimulation (15 trains of 4 pulses at 100 Hz every 200 ms) with a 30 s interval when repeated. Some recordings were carried out in presence of 25 µM picrotoxin, a GABA_A_ receptor blocker, in the perfusate. The magnitude of the effects on synaptic transmission was determined by measuring slopes of fEPSPs, which were modified in a similar way to fEPSP amplitudes, as reported by others also using MEA recordings [26]. Data are presented as means ± SEM on graphs plotting pooled data. Each individual (‘n’) corresponds to an experiment performed on a slice from an individual animal.

### Statistics

Significant differences between groups were determined by either one-way ANOVA on repeated measures for synaptic plasticity experiments and by one-way ANOVA on ranks for the quantification of Aβ deposits. The statistical significance of the difference between WT and 5xFAD mice when generating the I/O relationship was evaluated by performing a two-way ANOVA. Values represent the means ± SEM of the indicated number of independent experiments, and the level of significance was settled at p <0.05 (SigmaStat, Jandel).

## Results

**Figure 1:**
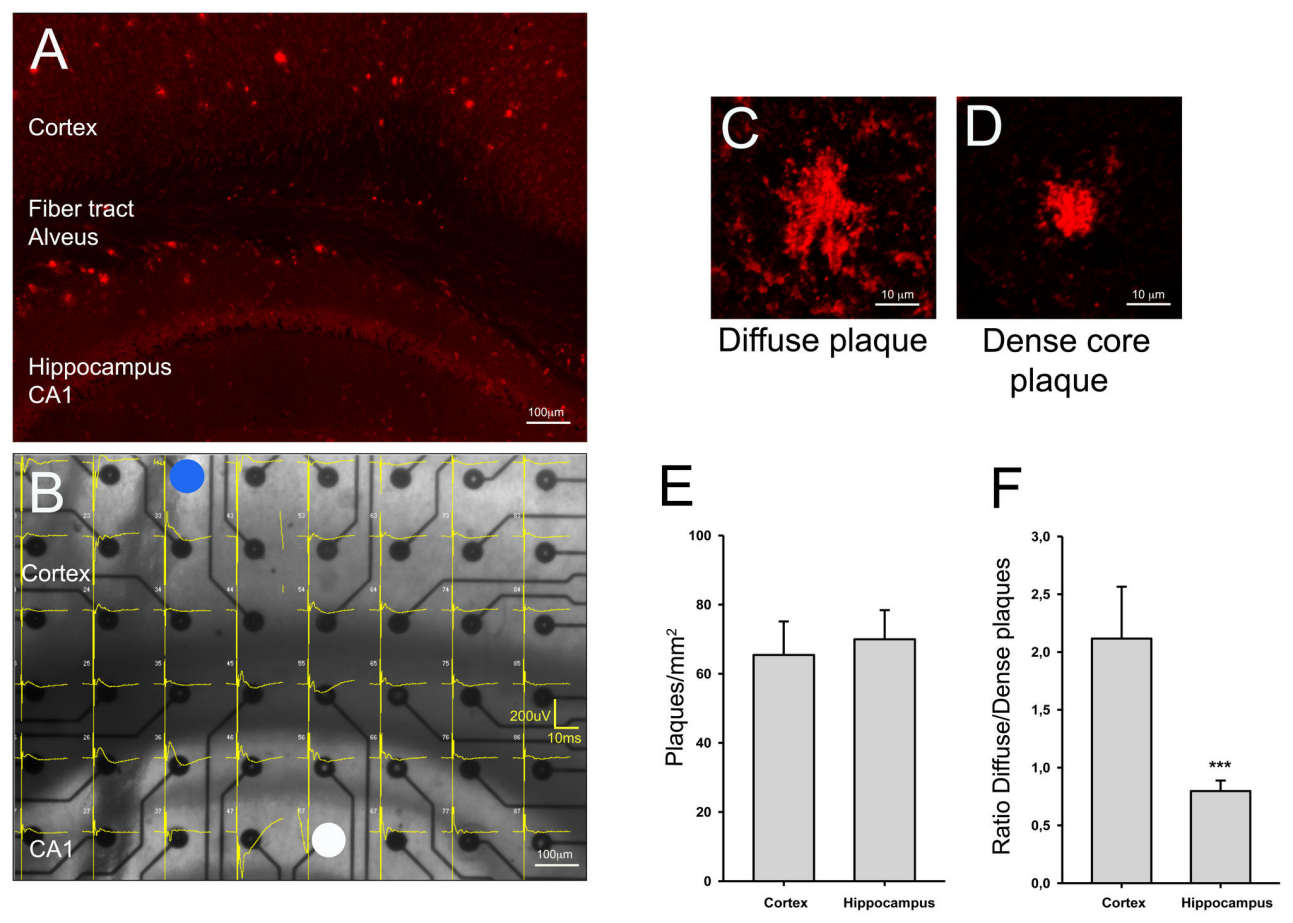
Amyloid deposition and recordings of synaptic transmission with MEA in brain slices of 5xFAD mice. (A) Immunostaining of Aβ peptide (anti-Aβ 6E10, red) and labeling of cell nuclei (with Hoechst, blue) in a brain section of a 6-month-old 5xFAD mouse. Inset: higher magnification image of a dense plaque with diffuse corona (top) and of a dense plaque without diffuse corona (bottom). (B) Synaptic signals evoked in the different areas of the brain slice. The captured image shows the brain slice positioned on the electrode array. The image of the MEA depicting the synaptic signals (fEPSPs and pop spikes) was further superimposed with respect to each electrode’s coordinates and indicated by numbers with the format ‘row, column’ of the MC Rack software. Field EPSPs and population spikes were recorded in the hippocampus and the cortex upon simultaneous stimulation in Schaffer collateral pathway of CA1 area of the hippocampus (indicated by white circle) and in the layer 5 of the SSC (indicated by blue circle). (C) Representative example of a diffuse plaque. (D) Representative example of a dense plaque. (E) Quantification of total number of plaques of Aβ deposits per mm^2^ in the cortex and the hippocampus. (F) Histogram depicting ratios of the number of plaques with diffuse corona over the number of plaques exhibiting only a dense core in the cortex and the hippocampus. *** p<0.001 compared with ratio in the cortex (ANOVA on ranks).

Most of AD mouse models start to develop amyloid plaques around 6 to 12 months. Here, we used the 5xFAD mice that exhibited plaques as soon as 2 months [7]. At 6 months of age, we observed severe occurrence of Aβ deposits, which densities were similar in the cortex and the hippocampus (Figure 1A and 1E), representing 65 ± 10 and 70 ± 8 plaques per mm^2^ respectively. However, there was a marked difference between the somato-sensorial cortex (SSC) and the hippocampus when considering the nature of the Aβ deposits. All deposits were composed of dense cores, some of them were surrounded by a diffuse corona. In order to compare the proportion of each type of Aβ deposits, i.e diffuse plaques (Figure 1C) *versus* dense plaques (Figure 1D), in the SSC and the hippocampus, they were quantified in both areas. Approximately twice as much diffuse plaques than dense plaques were encountered in the SSC (ratio 2.1 ± 0.5; Figure 1F), while in the hippocampus both types of Aβ deposits were equally distributed (ratio 0.8 ± 0.1; Figure 1F).

In order to explore whether such phenotypical changes in amyloid plaques could be associated with differences in synaptic functions, we developed a particular protocol of double stimulations with MEA, which allowed us to simultaneously record synaptic transmissions in both the hippocampus and the SSC within the same brain slice (Figure 1B). Synaptic signals could be evoked by afferent stimulations simultaneously applied in the layer 5 of the SSC (for example, electrode 32, indicated by a blue circle, Figure 1B) and in the Schaffer collaterals tract of the hippocampus (for example, electrode 57, indicated by a white circle, Figure 1B). Signals occurred either as field excitatory post synaptic potentials (‘fEPSPs’) - as observed for instance on electrode 37 in the *stratum radiatum* of the hippocampus and on electrode 22 in the layer 5 of the SSC in Figure 1B - or as population spikes (‘pop spikes’), as observed on electrode 36 in the *stratum oriens* of hippocampus and #24 in layer 6 of the SSC in Figure 1B. Mixed signals of both fEPSP and pop spikes could be recorded essentially in the *Alveus* (such as on electrode 45 in Figure 1B). Signals recorded in this last area have been discarded from further analyses.

**Figure 2:**
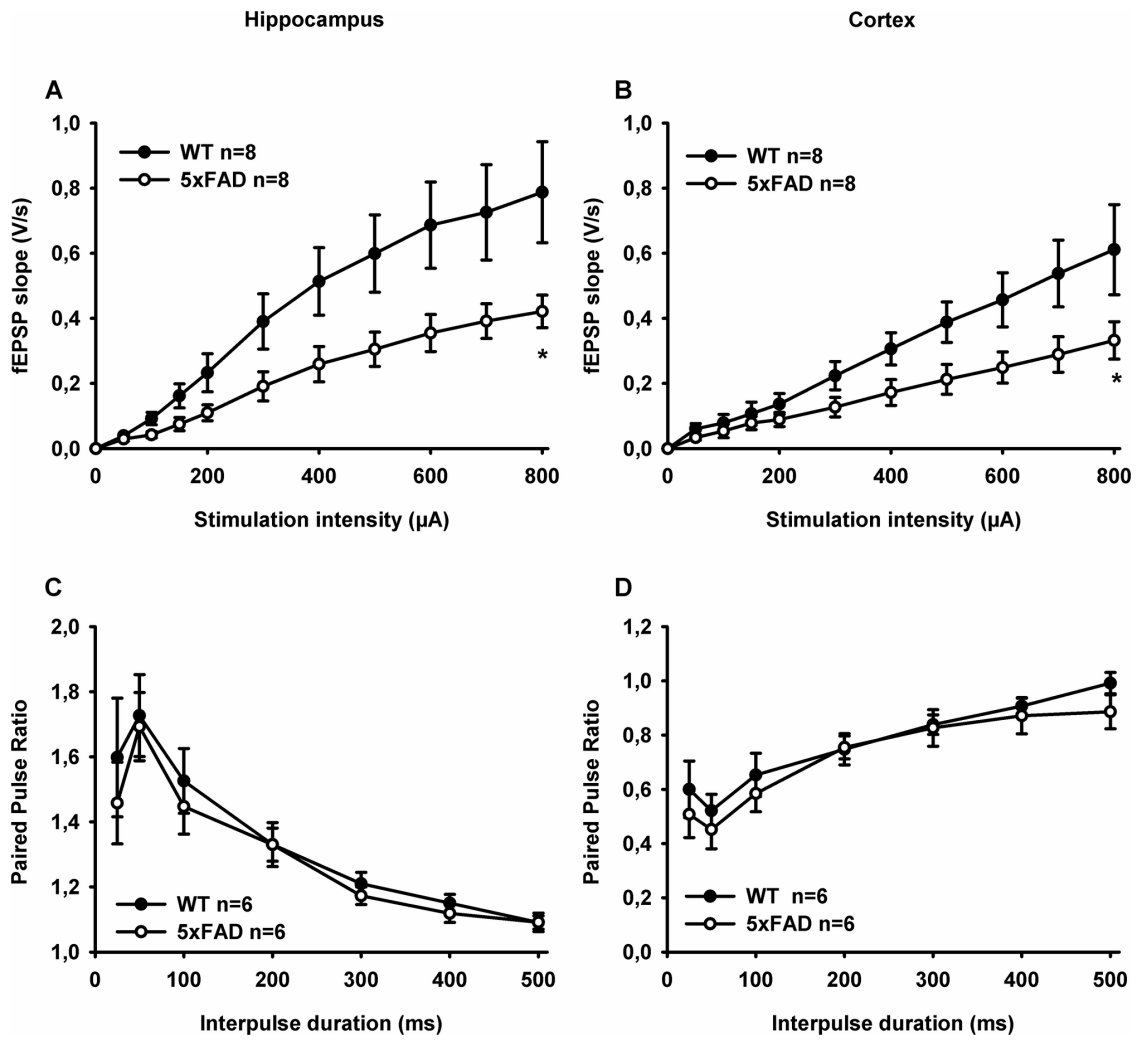
Basal synaptic transmission and short-term plasticity in hippocampal and cortical slices from WT and 5xFAD mice. (A and B) Input/output (I/O) curves obtained by plotting the slope of fEPSPs in the CA1 area of the hippocampus (A) and the layer 5 of SSC (B) of WT and 5xFAD mice as a function of the stimulation intensity (from 50 to 400 µA). * p<0.001 when comparing basal fEPSPs slopes recorded in WT mice with 5xFAD mice. (C and D) Paired-pulse ratio recorded across different inter-stimulus intervals (25–500 ms). Paired-pulse facilitation was observed in the hippocampus (C) and by contrast, paired-pulse depression was observed in the SSC (D) from WT and 5xFAD mice.

First, basal transmission and short-term synaptic plasticity were evaluated in 5xFAD mice and compared to those obtained in WT mice. In brain slices from 6 month-old 5xFAD mice, the relationships between fEPSP slope and stimulation strength (Input/Output (I/O) curve) were significantly different from those obtained in WT mice, either in the hippocampus (F_(1-132)_=23.58; p<0,001) or in the cortex (F_(1-132)_=21.08; p<0,001) (Figure 2A and 2B). This suggested that basal synaptic transmission in 5xFAD mice was affected at that stage in these brain areas.

Short-term plasticity in hippocampal and cortical synapses was assessed by measuring paired pulse ratio in hippocampus and cortex. Short-term changes, either facilitation or depression of synaptic strength, could be obtained by delivering paired stimuli with various intervals. In fact, paired-pulse facilitation could usually be triggered at hippocampal Schaffer collateral-CA1 synapses [27]. Such facilitation was observed in both WT and 5xFAD mice (Figure 2C). Varying the inter-pulse durations led to indistinguishable profiles of paired-pulse facilitation ratios in both WT and 5xFAD mice: from 50 ms, paired-pulse facilitation declined as far as interpulse duration increase (Figure 2C). By contrast, in the cortex, it have been described that paired-pulse stimulations led to short-term depression of fEPSPs [27]. Here we actually found similar paired-pulse induced depression in the cortex of both WT and 5xFAD mice (Figure 2D). The paired pulse ratio profile was characterized by a decline of depression as far interval duration increased (Figure 2D). Thus short-term plasticity in hippocampal and cortical excitatory synapses was unaltered in 5xFAD mice.

**Figure 3:**
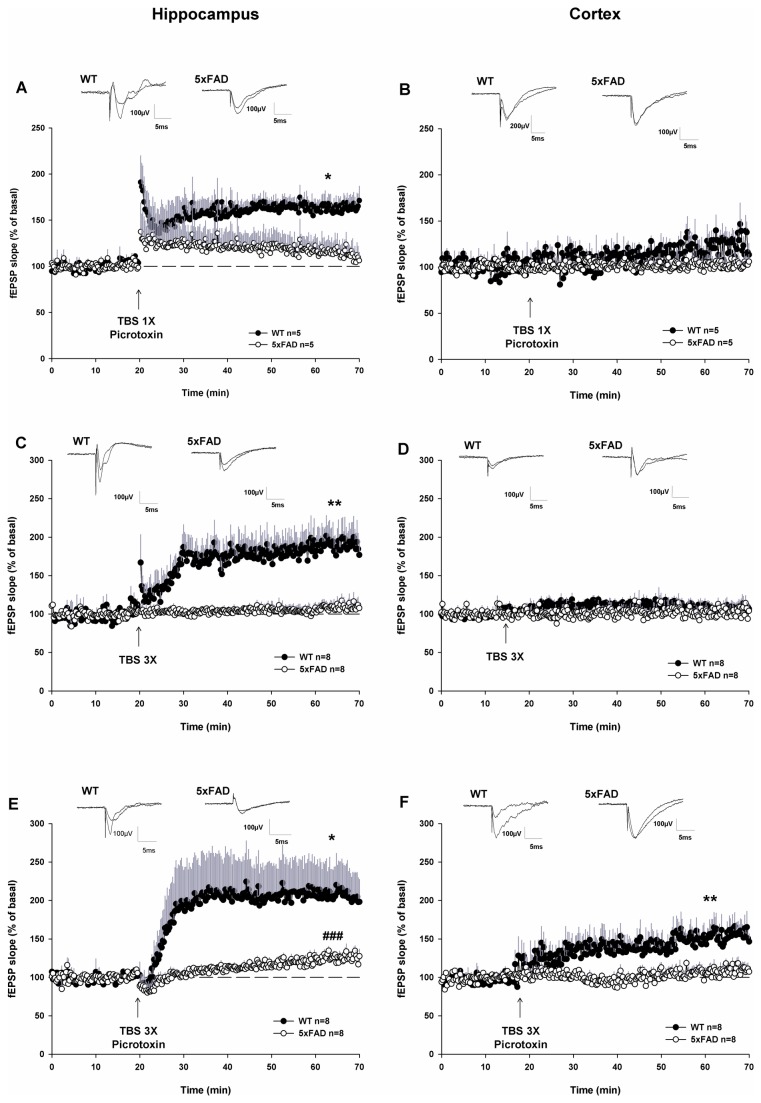
Impairment of LTP in cortico-hippocampal slices from 5xFAD mice triggered by different protocols of induction. (A and B) One theta burst stimulation (TBS) protocol applied in the presence of picrotoxin; this protocol induces LTP in the hippocampus of WT but not of 5xFAD mice (A). No LTP is observed in the cortex of WT and 5xFAD mice under these conditions (B). (C and D) Three TBS in the absence of picrotoxin; this protocol triggers LTP in the hippocampus of WT but not of 5xFAD mice (C). This protocol does not induce LTP in the cortex of either WT or 5xFAD mice (D). (E and F) Three TBS in the presence of picrotoxin; this protocol induces a robust LTP in hippocampus of WT mice and is largely attenuated in 5xFAD mice (E). Under these conditions, an enduring potentiation of fEPSPs is also obtained in the SSC of WT but not in the SSC of 5xFAD mice (F). On the graphs, data dots are fEPSP slopes normalized to their respective averaged baseline values recorded before TBS. Illustrative examples of fEPSPs recorded before and after TBS delivery are shown above each corresponding graph. * p<0.05 and ** p<0.01 compared with fEPSPs recorded in 5xFAD mice, # p<0.05 compared with basal fEPSPs of 5xFAD mice compared with one-way ANOVA.

Then we investigated whether the phenotypic difference in Aβ deposits between the CA1 area of the hippocampus and layer 5 of the SSC could be related to some different changes in long-term synaptic plasticity between these brain structures. For this, we evaluated the induction of long-term potentiation (LTP) in both the hippocampus and the cortex of WT and 5xFAD mice. We started by applying the classical protocol of high frequency stimulation (100Hz, 1s). Under these experimental conditions, LTP could not be triggered either in the hippocampus or in the cortex of both WT and 5xFAD mice (data not shown). This result could be explained by the strain of mice used to generate transgenic animals; mice with the B6/SJL genetic background used here being unable to develop any LTP under this classic protocol in our study. Indeed, the genetic background of mice was known to notably impact the induction and the maintenance of long-term plasticity, as well as of memory processes [28-30]. It has been previously demonstrated that LTP could be induced in the CA1 area of 5xFAD mice by applying “theta burst stimulus”: this protocol was consisting of 3 or 10 bursts of 4 pulses at 100 Hz, each burst being spaced out by a 200 ms inter-burst interval [6]. We used here a slightly modified protocol where theta burst stimulation comprised 15 bursts either 3 times repeated every 30 s (named TBS 3X) or not repeated (TBS 1X), in the presence or in absence of GABA_A_ receptor blocker, picrotoxin. In the absence of picrotoxin, TBS 1X did not induce any LTP either in the hippocampus or in the cortex of both WT and 5xFAD mice (data not shown). However, in the presence of picrotoxin TBS 1X was able to trigger a robust LTP in the hippocampus of WT mice; fEPSP slope increased to 165 ± 8% of basal, as measured 40 min after TBS 1X (Figure 3A). This LTP was fully abolished in the hippocampus of 5xFAD mice since no potentiation was observed: 40 min after TBS 1X, fEPSP slope was 123 ± 12% of the baseline slope (Figure 3A). By contrast, under the same experimental conditions, TBS 1X in the presence of picrotoxin, no LTP was detectable in the cortex even in WT mice (Figure 3B). Repeating TBS three times (TBS 3X) in the absence of picrotoxin gave results similar to those observed after applying TBS 1X in the presence of picrotoxin: in the hippocampus, 40 min after TBS 3X, fEPSP slope was increased to 175 ± 20% of basal slope in WT mice while fEPSP slope remained at 108 ± 7% of basal in the 5xFAD mice (Figure 3C). Here again, this protocol did not evoke any LTP in the cortex either in WT or in 5xFAD mice (Figure 3D). Nevertheless, TBS 3X in the presence of picrotoxin was the only paradigm tested able to trigger a robust LTP in both the hippocampus and the cortex of WT mice: under these experimental conditions, 40 min after TBS 3X, fEPSP slope rose to 206 ± 39% and at 146 ± 10% of basal slope in the hippocampus and SSC of WT mice, respectively (Figure 3E and 3F); these degrees of LTP being not significantly different from each other. In the hippocampus of 5xFAD mice, this LTP was drastically reduced; fEPSP slope was only enhanced to 126 ± 4% of basal, but remained significant (Figure 3E). Conversely, such LTP was fully abolished in the SSC of 5xFAD mice: indeed, fEPSP slope remained at 105 ± 8% of basal (Figure 3F).

## Discussion

The 5xFAD transgenic mouse model recapitulates in an accelerated fashion many pathological features of AD compared to other transgenic models [7,8]. They include Aβ deposition and neuroinflammation at 2 months of age in hippocampal and cortical areas followed by degeneration of synapses by 9 months of age as evidenced by reduction in synaptic marker proteins and pronounced loss of pyramidal neurons in cortical layer 5 and subiculum [7]. Such an early and robust phenotype is scarcely observed in other AD mouse models. As defined by Braak’s neuropathological staging, AD markers reach the hippocampus and the neocortex at stages III-IV and stages V-VI respectively [31]. However, the SSC could be affected earlier than previously described by Braak during AD progression since cerebral blood volume was decreased in this region of AD patients [32]. In addition, abnormal responses in the SSC were observed in patients with mild cognitive impairment [33]. Hippocampal plasticity has been largely studied in AD transgenic mouse model as reviewed by Marchetti et al. [34]. By contrast, few studies have examined cortical plasticity, although Battaglia et al. have shown a reduction of long-term potentiation in the cortex of APP/PS1 mice [35]. In this context, we have chosen to examine the electrophysiological characteristics of 5xFAD transgenic mice model at 6 months of age in both CA1 area of the hippocampus and layer 5 of the SSC simultaneously using an original method of double stimulation of each of these structures with MEA.

First, we compared basal transmission in 6 month-old 5xFAD mice and their WT littermates. Here, alteration of basal AMPA receptor-mediated synaptic transmission in 5xFAD mice was observed in both the hippocampus and the SSC. This observation is in agreement with previous studies showing a reduction of hippocampal basal transmission in 6 month-old 5xFAD mice, although no difference was observed at 4 months of age [6,36].

Next, we have examined short-term plasticity by studying paired-pulse ratio in the CA1 area of hippocampus and layer 5 of the SSC. This protocol induces synaptic changes, either facilitation or depression of synaptic strength. The paired-pulse facilitation observed in CA1 area of the hippocampus is usually attributed to effects of residual elevation of calcium concentration at presynaptic sites, while paired-pulse depression observed in layer 5 of the SSC is mainly associated with depletion of the pool of readily releasable vesicles [27]. No modification of short-term plasticity was detectable in both hippocampus and cortex of 5xFAD mice as compared to WT mice. With regard to the hippocampus of 5xFAD mice, this result is in agreement with other studies [6]. Moreover, studies on short-term plasticity in the hippocampus of other APP transgenic mouse models led to similar conclusion [37-39]. Therefore, we have highlighted that presynaptic sites seem to be spared in the hippocampus and in the SSC of the 5xFAD mice.

Finally, we examined synaptic plasticity induced by TBS in the CA1 area of the hippocampus and the layer 5 of the SSC. TBS is an LTP induction protocol which mimics endogenous theta frequency of EEG activity recorded in the hippocampus during behavioral activity [40]. In the hippocampus of WT mice, similar LTP were observed using either one or three trains of (TBS 1X and 3X). This finding suggests that the potentiation is saturating in this structure. In contrast, in the SSC of WT mice, under the same experimental conditions, LTP was only induced by TBS 3X but not by TBS 1X. This result is in agreement with the fact that the magnitude of LTP is matched in stimulus number in this cerebral structure [41,42]. We find here that long-term synaptic plasticity is completely abolished in the SSC, but still detectable, although drastically reduced, in the hippocampus of 5xFAD mice. Indeed, even when applying a ‘strong’ stimulation protocol (TBS 3X with picrotoxin) which triggers a robust LTP in the cortex of WT mice, no LTP could be induced in the cortex of the 5xFAD mice. When considering Aβ immunolabelling in 6 month-old 5xFAD mice in the cortex and the hippocampus, we observed an inverse correlation between the occurrence of long-term synaptic plasticity and the proportion of Aβ depositions with diffuse corona in these areas; Aβ deposits with diffuse corona being prominent in the cortex at that stage. Therefore the rate of diffuse corona around the dense core of Aβ deposits matches the extent of the synaptic plasticity losses. This is consistent with the fact that the diffuse corona contains swollen distended neurites that may indicate neuronal injury [2]. Moreover, the presence of synaptic vesicles [43] could suggest that aberrant connections are formed as previously demonstrated in the cortex where AD preferentially disorganized the cortico-cortical circuitry [44]. The loss of LTP could also be attributed to a pronounced exposure of cortical neurons to Aβ that alters the activity of synaptic AMPA and NMDA receptors. First of all, we observe, in 5xFAD mice, a decreased basal synaptic transmission which is supported by AMPA receptors. Many reports show that Aβ targets AMPA receptors by promoting their endocytosis likely *via* the activation of dephosphorylation pathways which may involve α-7 nicotinic acetylcholine receptors [45,46]. This may cause modifications of neuronal circuitry. Otherwise, the LTP impairment that we observed could be due to a direct agonistic action of Aβ on NMDA receptors [47]. Alternatively, this loss of cortical plasticity could be associated with a profound reorganization of cortical networks [48]. We found that cortical LTP can be triggered only when GABA_A_ receptor is blocked with picrotoxin, as evidenced earlier (Hess et al., 1996). In this view, several studies have recently highlighted an alteration of inhibitory interneurons in AD and in transgenic mouse models [49-52]. A transient increase followed by an irreversible decrease in the number of glutamatergic and GABAergic presynapses was correlated with the progression of AD [53,54]. Thus, the fact that we do not observe any LTP in the SSC of 5xFAD mice in the presence of picrotoxin could be due to a reduction in the GABAergic tone, and a subsequent remodeling of synaptic contacts, enabling normal basal synaptic transmission but not long-term plasticity of synaptic transmission.

The complete loss of LTP in the cortex is in accordance with the impairment of behavioral tasks observed in the 5xFAD mice. In fact, in 6 month-old 5xFAD mice, the loss of cortical plasticity could be at the origin of the defects of cortex-dependent behaviors as evaluated with different tests such as the H-maze [11], the contextual fear conditioning [6], or the conditioned taste aversion [10]. More precisely, Girard et al. have reported that early cognitive deficits related to frontal cortex occurred in 4-month-old 5xFAD mice before impairments of hippocampal-dependent learning and memory could be observed [6]. These conclusions are in complete agreement with our present study suggesting that cortical plasticity seems to be more impaired than hippocampal plasticity in 6 month-old 5xFAD mice. In summary, even if some of these behavioral tasks usually involve not only cortical areas but also other brain regions like the hippocampus or the amygdala, we conclude that cortical plasticity is totally deficient in the 5xFAD model which correlates with the prominent presence of the Aβ dense core deposits surrounded by diffuse corona. The methodology used in this study which allows to record simultaneously many brain areas could be useful for a better understanding of area-specific molecular mechanisms implicated in neuropathologies. It could contribute to the development of more targeted therapeutic strategies.
